# Rational Design of Hyaluronic Acid-Based Copolymer-Mixed Micelle in Combination PD-L1 Immune Checkpoint Blockade for Enhanced Chemo-Immunotherapy of Melanoma

**DOI:** 10.3389/fbioe.2021.653417

**Published:** 2021-03-10

**Authors:** Chaopei Zhou, Xiuxiu Dong, Chunxiang Song, Shuang Cui, Tiantian Chen, Daji Zhang, Xiuli Zhao, Chunrong Yang

**Affiliations:** ^1^College Pharmacy, Jiamusi University, Jiamusi, China; ^2^School of Pharmacy, Shenyang Pharmaceutical University, Shenyang, China

**Keywords:** hyaluronic acid, mixed micelle, chemo-immunotherapy combinations, drug delivery system, programmed cell death ligand 1 (PD-L1)

## Abstract

The application of combinational therapy breaks the limitation of monotherapy and achieves better clinical benefit for tumor therapy. Herein, a hyaluronic acid/Pluronic F68-based copolymer-mixed micelle was constructed for targeted delivery of chemotherapeutical agent docetaxel (PHDM) in combination with programmed cell death ligand-1(PD-L1) antibody. When PHDM+anti-PDL1 was injected into the blood system, PHDM could accumulate into tumor sites and target tumor cells *via* CD44-mediated endocytosis and possess tumor chemotherapy. While anti-PDL1 could target PD-L1 protein expressed on surface of tumor cells to the immune checkpoint blockade characteristic for tumor immunotherapy. This strategy could not only directly kill tumor cells but also improve CD8^+^ T cell level and facilitate effector cytokines release. In conclusion, the rational-designed PHDM+anti-PDL1 therapy strategy creates a new way for tumor immune-chemotherapy.

## Introduction

Cancer is a great threat to human health, and chemotherapy has been widely applied in clinics (Wu et al., 2016; Zheng et al., 2017; Cheng et al., 2018; Jiang et al., 2019; Dai et al., 2020; Zhou F. et al., 2021). However, intravenous administration of a free chemotherapeutical agent for tumor therapy would bring severe systematic toxicity and side effects (Li et al., 2017; Luo et al., 2018; Han et al., 2019; Liu et al., 2019; Shi et al., 2019). The development of nanoparticle drug delivery systems (NDDS) could not only reduce drug nonspecific distribution but also increase tumor accumulation *via* the enhanced permeability and retention (EPR) effect (Zhang et al., 2017, 2018a,b, 2019). However, a major problem lies in the insufficient tumor target capability, which resulted in unsatisfied antitumor efficacy. Hyaluronic acid (HA) is a polyanionic polysaccharide, which is biocompatible and safe for intravenous administration (Huo et al., 2017; Trujillo-Nolasco et al., 2019; Razavi and Huang, 2020). Besides this, HA has been widely used for the construction of tumor-targeting nanoparticles because it could specifically target CD44 receptors of tumor cells and facilitate cellular uptake (Huo et al., 2017; Cai et al., 2019; Zhang et al., 2020; Rangasami et al., 2021). The copolymer micelle is an amphiphilic molecule, which could self-assembly into a nanoparticle in aqueous solution (Zhang et al., 2016, 2017). Structure-modified HA with hydrophobic molecules could form grafted amphiphilic copolymers and be used for micelle construction. Vitamin E succinate (VES), as a natural hydrophobic vitamin E derivative, could not only serve as hydrophobic residue, but also possess potential antitumor activity (Fan et al., 2021; Farooq et al., 2021; Puig-Rigall et al., 2021). Therefore, the HA-*g*-VES-grated copolymer is suitable for copolymer micelle construction.

Despite many advantages of copolymer micelles in tumor targeting and tumor therapy, mono-grafted micelles possessed unsatisfied stability, which might induce drug leakage and dissociation of micelles. Therefore, the introduction of other copolymers and the construction of mixed micelles is a promising strategy for enhancing the stability. Pluronics as triblock copolymers with repeated a PEO-PPO-PEO sequence has attracted great attention for NDDS due to its biocompatibility and biodegradability, and Pluronic F68 has been widely used for construction of temperature-sensitive gels (Huang et al., 2008; Al Khateb et al., 2016; Powell et al., 2017; Patil et al., 2019; Wang et al., 2019). The introduction of F68 in copolymer micelles is a promising strategy for enhancing the stability of the copolymer micelle. Docetaxel (DTX) is an active derivative of paclitaxel. DTX can inhibit the depolymerization of microtubules, ultimately resulting in the loss of function of forming microtubule bundles and thereby inhibiting the mitosis of cancer cells (Rafiei and Haddadi, 2019; Dawoud et al., 2020; Ertugen et al., 2020; Li et al., 2021). Therefore, DTX was used to incorporate into the copolymer mixed micelle for tumor chemotherapy.

Despite that chemotherapy could directly reduce tumor volume and increase the survival rate, drug resistance and severe side effects remain unsatisfactory (Shen et al., 2014; Fan et al., 2018; Zhang et al., 2018b). Hence, a combination with antibodies, inhibitors, or sensitizers seems to be a prosing strategy for enhance tumor therapy efficacy. Immunotherapy emerges as a promising strategy for enhance antitumor activity (Qin et al., 2020; Zhou W. et al., 2021). An immune checkpoint inhibitor based on programmed cell death ligand 1 (PD-L1) antibodies is a promising breakthrough for tumor therapy. Anti- PD-L1 could activate CD8^+^ T cells and inhibit regulatory T cells, which results in the effector cytokines effect (Choi et al., 2019). Although there are many advantages for PD-L1-based immunotherapy, a low response rate is a critical problem. It has been reported that combination chemotherapy and immunotherapy could make the tumor sensitive to chemotherapeutics and respond to immunotherapy, which possesses a better synergistic antitumor effect (Yang et al., 2019).

**Scheme 1:**
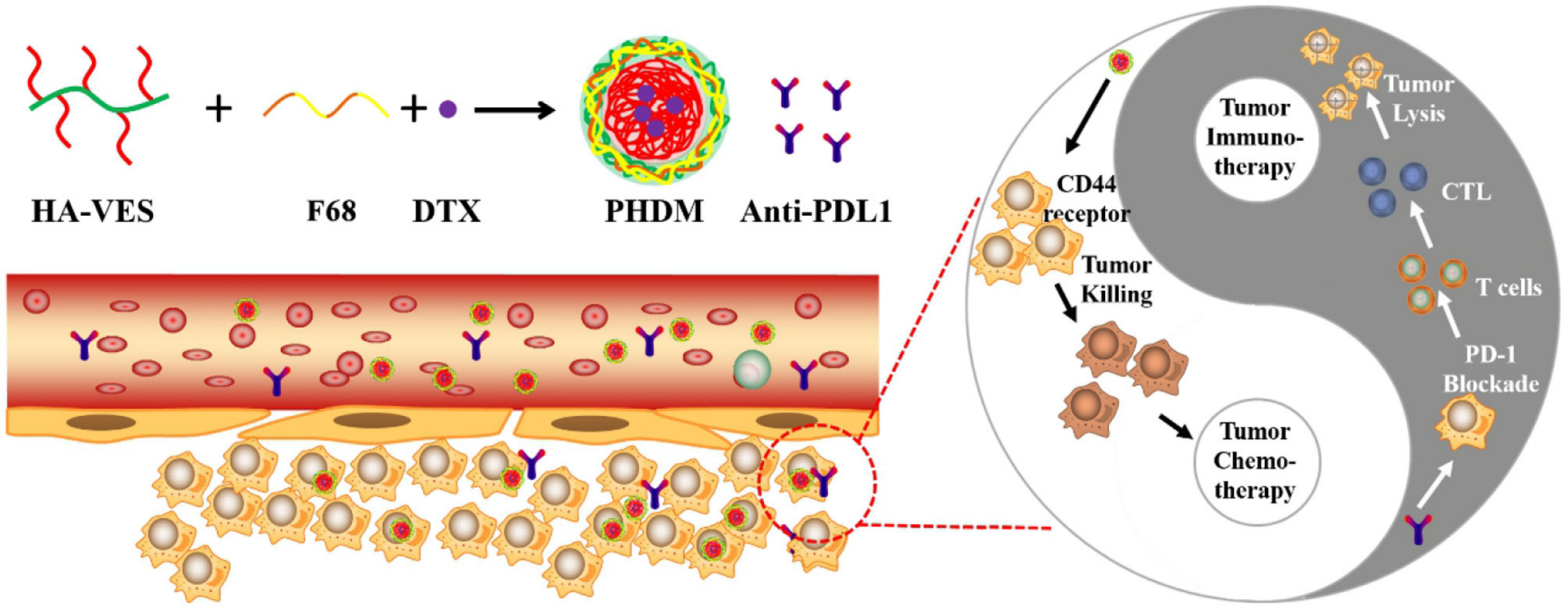
Schematic illustration of construction of PHDM and the potential mechanism for tumor immune-chemotherapy for PHDM+anti-PDL1.

In this work, we synthesized the HA-VES grafted copolymer, and DTX was selected as a model drug for construction of grafted copolymer micelle Hyaluronic acid-based Docetaxel Micelle (HDM). Meanwhile, F68 was introduced into the copolymer micelle and the prepared mixed micelle to enhance *in vitro/vivo* stability Pluronic modified Hyaluronic acid-based Docetaxel Micelle (PHDM) and in combination of PD-L1 antibody to possess tumor chemo-immunotherapy (Scheme 1). This strategy demonstrated superior tumor-targeting efficacy and tumor suppression.

## Method and Materials

### Materials, Cells, and Animals

HA (MW = 10,000) was purchased from Bloomage Freda Biopharm Co. Ltd (Shangdong, China). 1-Ethyl-3-(3-dimethylaminopropyl) carbodiimide (EDC), N-hydroxysuccinimide (NHS) was purchased from Shanghai Civi Chemical Technology Co., Ltd. (Shanghai, China). VES was obtained from Jiangsu XiXin Vitamin Co., Ltd. (Jiangsu, China). Pluronic F68 was purchased from Sigma Chemical Co., Ltd. (St. Louis, MO). DTX was acquired from Shanghai Jinhe Biotech Co., Ltd. (Shanghai, China). Dialysis bag (MW = 3,500) was obtained from Union Carbide Corporation. (Danbury, CT USA). Dulbecco's Modified Eagle's Medium (DMEM) was purchased from Thermo Fisher Scientific Inc. (Waltham, MA USA). Fetal bovine serum (FBS) was acquired from Tianjin Kangyuan Biotechnology Co., Ltd. (Tianjin China). Cell-counting kit-8 (CCK-8) was obtained from Nanjing KeyGen Biotech Co., Ltd. (Naijing, China).

B16-F10 cells were obtained from American Type Culture Collection (ATCC, Manassas, VA, USA) and cultured in DMEM with 10% FBS and 1% penicillin-streptomycin (Gibco) at 37°C under 5% CO_2_ atmosphere.

C57BL/6 mice (6–8 weeks) and BALB/c mice were purchased from Beijing HFK Bioscience Co., Ltd. (China), and B16 cell suspension (about 2 × 10^7^ cells in 75 μL of PBS) was subcutaneously injected into the left flank of mice, and when the tumor volume reached ~100 mm^3^, they were divided into several groups for further use. All the animal experiments were carried out according to the guidelines of the Experimental Animal Administrative Committee of Jiamusi University.

### Synthesis of HA-VES

HA (0.02 mmol) was dissolved in 10 mL formamide at 50°C to complete dissolved and cooled to room temperature, subsequently, EDC (0.22 mmol), DMAP (0.22 m mol), and VES (0.2 mmol) were added into the mixture, and the mixture was stirred at room temperature overnight. The mixture was dialyzed by a dialysis bag (MWCO 3,500 Da) for 48 h. Finally, the solution was filtered, and lyophilized and HA-VES was obtained. (~1.92 ppm 3.23~4.67 ppm methyl proton of N-acetyl group, 3.23~4.67 ppm methylene and hydroxyl groups of HA; 1.51~1.81 ppm and 2.02~2.24 ppm methyl and methylene protons of VES).

### CMC Measurement

Critical micelle concentration (CMC) of HA-VES was measured by pyrene fluorescent probe. Briefly, pyrene was 6 × 10^−8^ mol L^−1^ was transferred into a brown flask, and a gradient concentration of HA-VES was added into each flask. Samples were under ultrasound in the water bath at 50°C for 2 h and stored overnight under dark conditions. Finally, the samples were measured by fluorescent spectrometer at 338 and 334 nm.

### Preparation of DTX-Loaded Micelles

The dialysis method was used to prepare DTX-loaded micelles. Briefly, 10 mg HA-VES/7 mg HA-VES + 3 mg F68 and 1 mg DTX were dissolved in DMSO and stirred at room temperature until completely dissolved. The mixture was transferred into a dialysis bag for dialysis and to remove organic solvent. Finally, the samples were filtered, and HDM and PHDM were prepared.

### Characterization of Formulations

The size and **ζ-**potential of the HDM or PHDM were measured by Zetasizer (Nano ZS90, Malvern Instruments Ltd.). The morphology of different formulations was observed using transmission electron microscopy (TEM; JEOLJEM-1230 microscopes at 120 kV, JEOL, Japan).

### Stability Measurement

*In-vitro* stability measurement was investigated, and particle size change of different formulations was measured. Briefly, both formulations (5 mL) were placed in shaking incubator at 37°C, 100 rpm. At an interval time point, 0.5 mL samples were taken for particle size determination. Meanwhile, encapsulation efficacy (EE, %) change was also measured by ultracentrifugation method. HDM or PHDM were dissolved in PBS (10 mL). The solution was centrifuged for 10 min, the filtrate (0.5 mL) was transferred into a 10-mL brown volumetric flask, and made up to the volume with methanol as the free DTX. Next, another unfiltered formulation was transferred into a 10-mL brown volumetric flask, and made up to the volume with methanol as the the total DTX, and calculate EE% as reference reported. For the stability assay, both formulations were placed in shaking incubator at 37°C. At an interval time point, 0.5 mL samples were taken for EE% measurement and particle size determination.

### *In vitro* Drug Release Study

The *in-vitro* release process of different formulations was examined using the dialysis method. Different formulations were transferred into dialysis bags (MWCO 3,500 Da), and the dialysis bags were immersed in a 100 mL phosphate buffer saline (PBS, pH = 7.4) with moderate shaking (50 rpm) at 37 ± 0.5°C. Fresh medium was used to replace released medium at the predetermined time points. The cumulative release was plotted against time.

### *In-vitro* Cell Cytotoxicity and Cell Apoptosis Assay

Cytotoxicity of different micelles was studied by the MTT method. In brief, B16 cells were seeded in 96 well plates at a density of 10^4^ cells and treated with various samples. At specified time intervals, 10 μL MTT solution was added into each well, and the plate was incubated for another 4 h. subsequently, the medium was removed and DMSO was added into each well. The plates were shaken and determine the absorbance at 450 nm was taken by the microplate reader.

The cell apoptosis assay was investigated with the Annexin V-FITC kit. The detailed operation procedure was according to the manufacturer of the kit.

### *In-vitro* Cell Uptake Assay

Nile red (NR) was used as a fluorescence probe to represent DTX and incorporated it into different formulations for a cellular uptake assay. Briefly, cells were seeded into a glass-covered six-plate well and allow attachment. Different formulations were added into each well and incubated for different times. At the interval time point, the medium was removed and washed with PBS, harvested with trypsin for flow cytometry, and counterstained with 4′,6-diamidino-2-phenylindole (DAPI) for Confocal laser scanning microscopy (CLSM) assay.

For investigating intracellular drug release, LysoTracker-Green was used to pre-stain intracellular endo/lysosomes. Subsequently, different formulations were added into each well and incubated for 4 h. The cells were fixed with formalin and stained nucleic with DAPI, visualized using CLSM.

### *In-vivo* Tumor-Targeting Study

To investigate *in-vivo* tumor-targeting of different formulations, DiR was used as fluorescence probe and incorporated into the micelles. Tumor-bearing nude mice were intravenously administrated different DiR-loaded micelles for circulation. At the interval time point, the mice were anaesthetized and visualized using an IVIS system. After 24 h, mice were sacrificed, and their major organs were obtained for imaging.

### *In-vivo* Antitumor Activity

B16 bearing mice were randomly divided for several groups (Control, DTX, HDM, PHDM, PHDM+anti-PDL1). All mice received intravenous administrated of different formulations every other day for four times and measured for the tumor volume and body weight change. At the end of the trail, tumor tissues were weighed, and blood was collected for further study.

### Cytokines Measurement

Serum TNF-α,IFN-γ,and IL-2 level were measured using an ELISA kit. The detailed procedure was according to the manufacture of the kit.

### Immune Cell Analysis

Freshly isolated tumor tissue was obtained and cut into small pieces. The tumors were digested with collagenase IV for 1 h. Subsequently, the cells were filtered to prepare a single-cell suspension. The cells were stained with a cocktail antibody for flow cytometry. CD3-FITC and CD8-PE were used to detect CD8^+^ T cells. CD4-FITC and Foxp3-PE were used to label Tregs.

### Statistical Analysis

All data expressed as mean ± standard deviations (SD). Significant different between two groups was analyzed using two-tailed Student's *t*-test.

## Results and Discussions

### Characterization of Different Formulations

**Figure 1:**
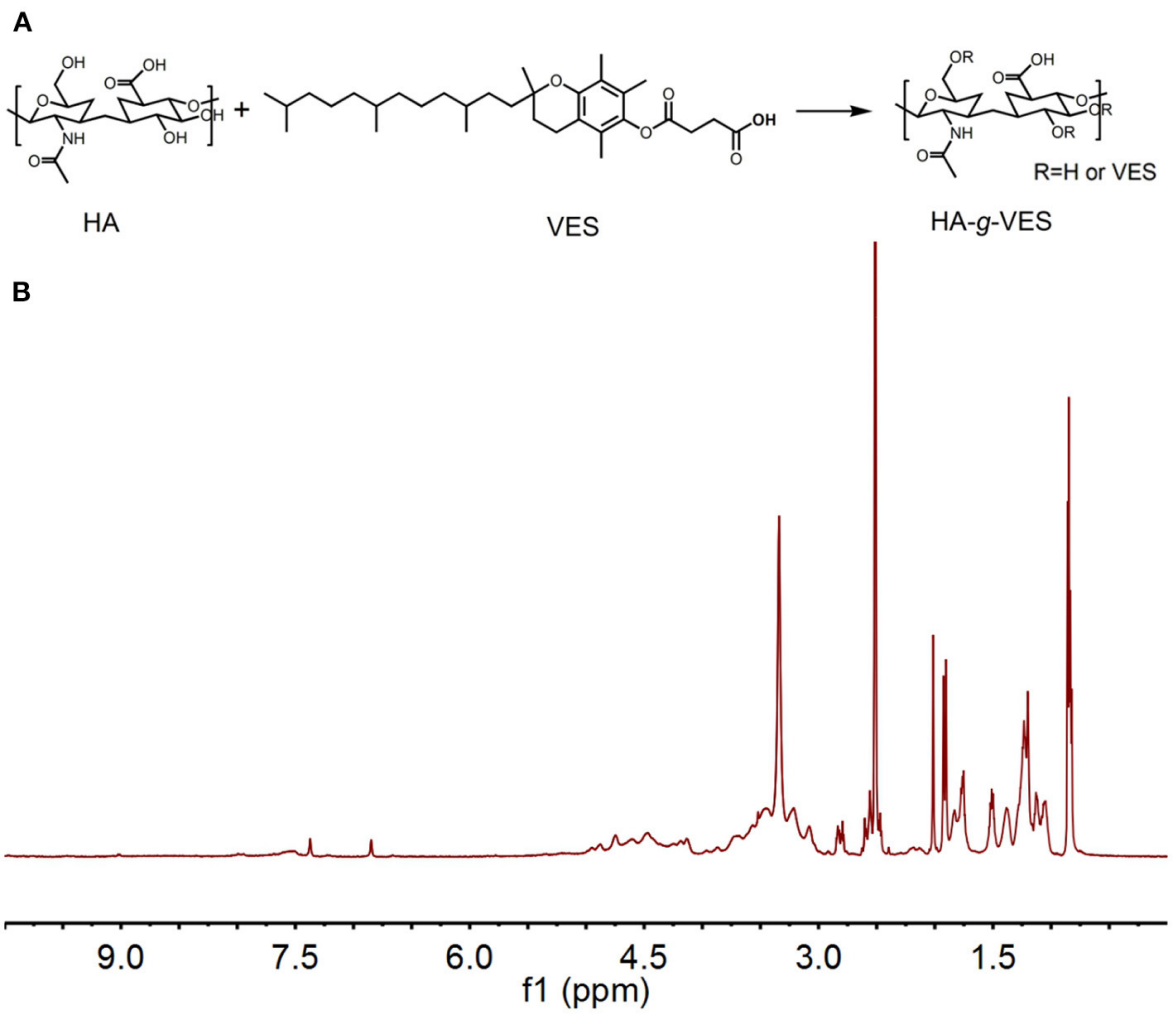
**(A)** Synthesis route of HA-VES **(A)** and **(B)** ^1^H NMR spectrum.

HA-VES was synthesized firstly *via* the ester reaction. Due to the presence of amount of hydroxyl groups in HA, it's easy to modify different functional backbones on the surface of HA through the ester reaction. Due to active carboxyl groups in VES and hydrophobic characteristic, it is suitable to construct HA-VES-grated amphiphilic copolymers, which could form micelle structures in an aqueous environment. The synthesis route and ^1^H NMR spectrum is illustrated in Figures 1A,B. The appearance of characteristic peaks (~1.92 ppm 3.23~4.67 ppm methyl proton of N-acetyl group, 3.23~4.67 ppm methylene and hydroxyl groups of HA; 1.51~1.81 ppm and 2.02~2.24 ppm methyl and methylene protons of VES) indicated HA-VES has been synthesized successfully.

**Figure 2:**
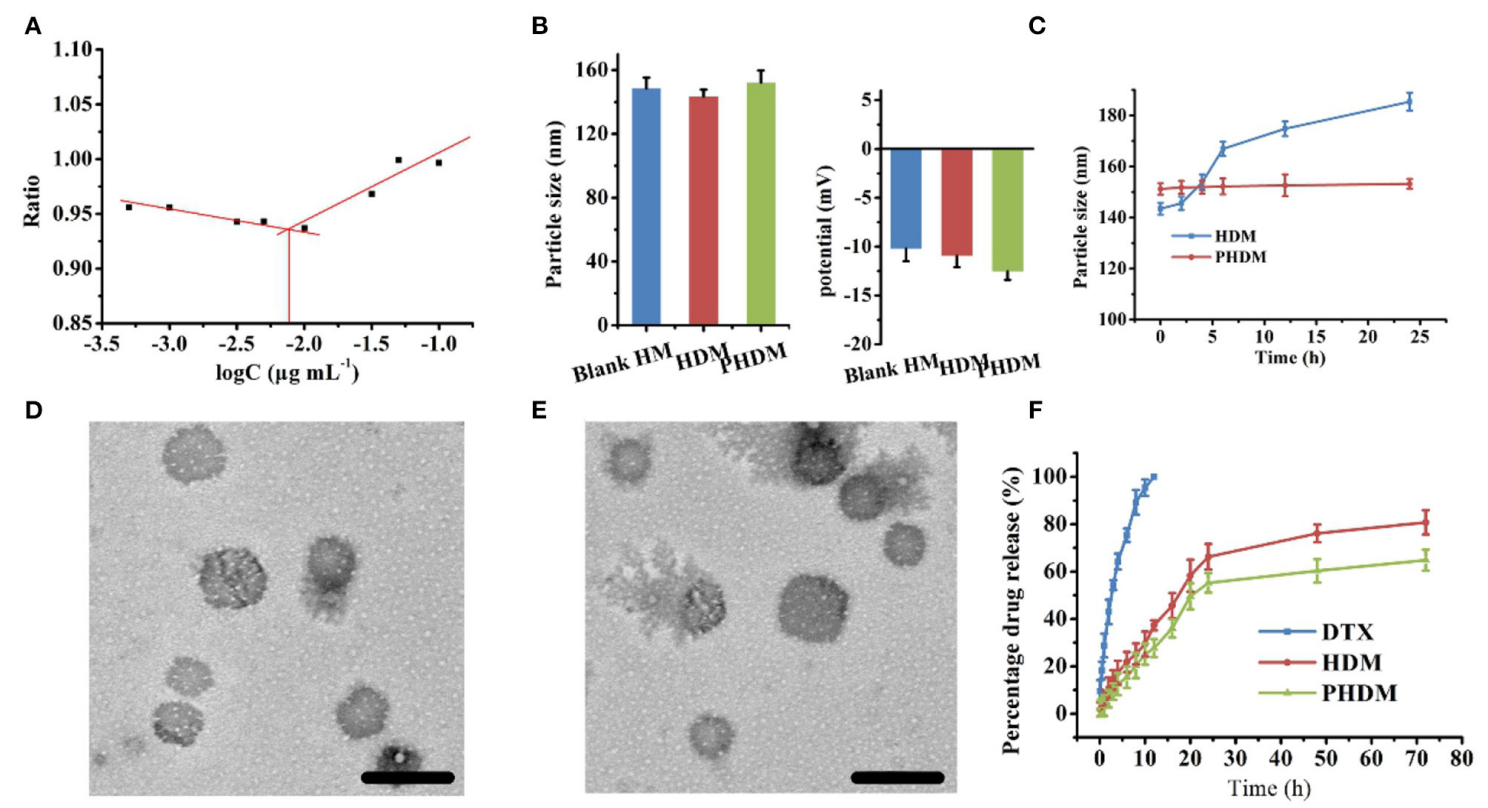
**(A)** CMC value of HA-VES copolymer measured with pyrene fluorescence method; **(B)** particle size and ζ-potential of different formulations; **(C)** *in vitro* stability of HDM and PHDM; **(D,E)** TEM images of HDM and PHDM, scale bars represented 200 nm; **(F)** *in vitro* drug-release profile of different formulations.

CMC is an important parameter for evaluating the aggregation capability of copolymer in aqueous condition, which influences the self-assembly ability and structural stability of copolymer micelles both *in vitro* and *in vivo* (Patil et al., 2019; Puig-Rigall et al., 2021). In this section, the CMC value of HA-VES was measured. As shown in Figure 2A, the CMC value of HA-VES was 6.6 mg L^−1^, which indicated that HA-VES could form micelles at extremely low concentrations. The relative low CMC value indicated a better structural stability, which could possess the integrity of the copolymer micelle in extreme dilution. Particle size and ζ-potential of different formulations were measured, as shown in Figure 2B; both HDM and PHDM possessed average particle size of ~130 nm with negative surface charge. The introduction of F68 brings a slight increase in particle size, further demonstrating the successful construction of mixed micelles. Subsequently, *in-vitro* stability of different formulations was measured, as shown in Figure 2C and Supplementary Material 1; particle size and EE% change were both investigated. With an increase of incubation time, particle size of HDM gradually increased from ~140 to ~180 nm, while there was no obvious change for PHDM. Meanwhile, EE% of HDM significantly decreased after 24-h incubation. Both results indicated that HDM was relatively unstable, and this phenomenon was mainly attributed to the leakage of DTX and would bring a decrease of hydrophobic interaction of micelle inner core, which resulted in a change of particle size. While there was no obvious change for PHDM, this result indicated that the presence of F68 would enhance hydrophobic interaction between the drug and carrier and enhance its physical stability, which would reduce non-specific drug release and reduce systematic toxicity. The morphology of both formulations were observed. As shown in Figures 2D,E, both HDM and PHDM were spherically shaped with uniform particle size, which was in consistent with Dynamic Light Scattering (DLS) data. *In-vitro* drug release profiles were investigated, and the result is shown in Figure 2F. Both formulation groups possessed sustained release behavior with the total drug release percentage at about 60%, which indicated both formulations were suitable for intravenous administration. Interestingly, PHDM possessed relative lower release rate compared with HDM, and this phenomenon was mainly attributed to the introduction of F68 that would bring a hydrophobic interaction between the drug and carrier and influence drug release from the carrier.

### Cell Cytotoxicity and Cell Apoptosis Assay

**Figure 3:**
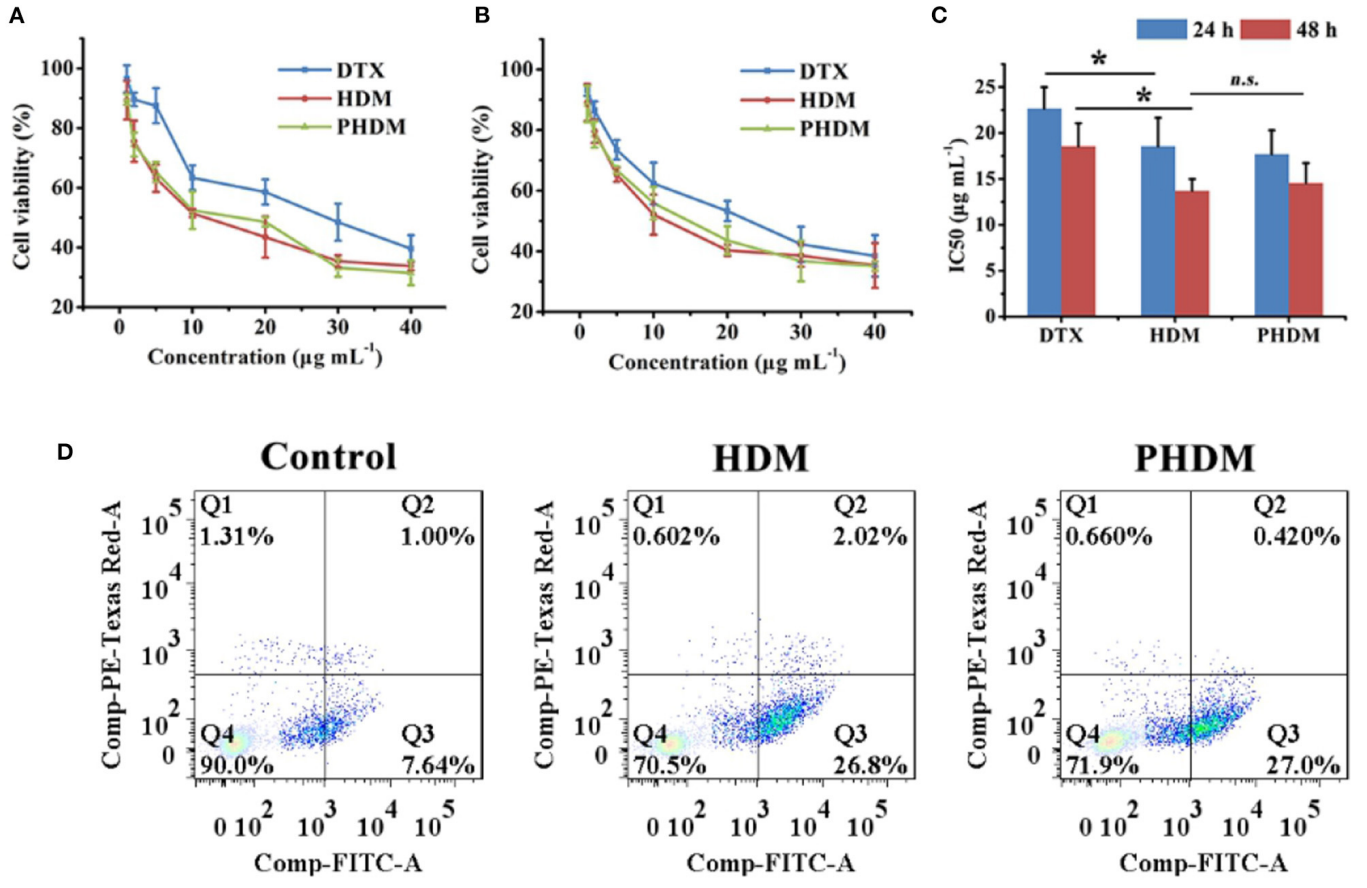
*In-vitro* cell cytotoxicity of different formulations at 24 h **(A)** and 48 h **(B)** and IC_50_ value **(C)** of different formulations using MTT method with B16 cell line; **(D)** *in vitro* cell apoptosis assay of different formulations with Annexin V-FITC/PI method.

*In-vitro* cell cytotoxicity of different formulations were measured using MTT method against the B16 cell line. As shown in Figures 3A,B, all groups possessed a significant time-dependent and dose-dependent cytotoxicity manner. Compared with DTX, HDM possessed higher cytotoxicity, and this result was mainly attributed to CD44-mediated endocytosis of the carrier, which facilitated intracellular drug accumulation. However, the free drug could only accumulate into tumor cells *via* passive diffusion, and the efficacy was limited. Meanwhile, no significant difference could be observed for both HDM and PHDM. Although F68 would form a hydrophilic shell on the surface of micelle, this shell was incomplete and could not totally block HA expose, only minor percentage of active targeting moiety could reach tumor-active targetability, which indicated F68 didn't influence the interaction between the CD44 receptor and HA residues.

Cell apoptosis was considered as an important symbol of programmed cell death, and it is necessary to investigate whether the cell cytotoxicity arose from apoptosis or necrosis. As shown in Figure 3C, the control group showed minor apoptosis cells, which indicated the cells were in a good state. After incubation with both HDM and PHDM, an obvious increase for apoptosis cells could be observed, indicating both formulations could efficiently induce tumor cell apoptosis. The cell apoptosis results of both micelle formulations possessed the similar trend as MTT, further demonstrating the cell cytotoxicity of as-prepared formulations.

### *In-vitro* Cell Uptake Assay

**Figure 4:**
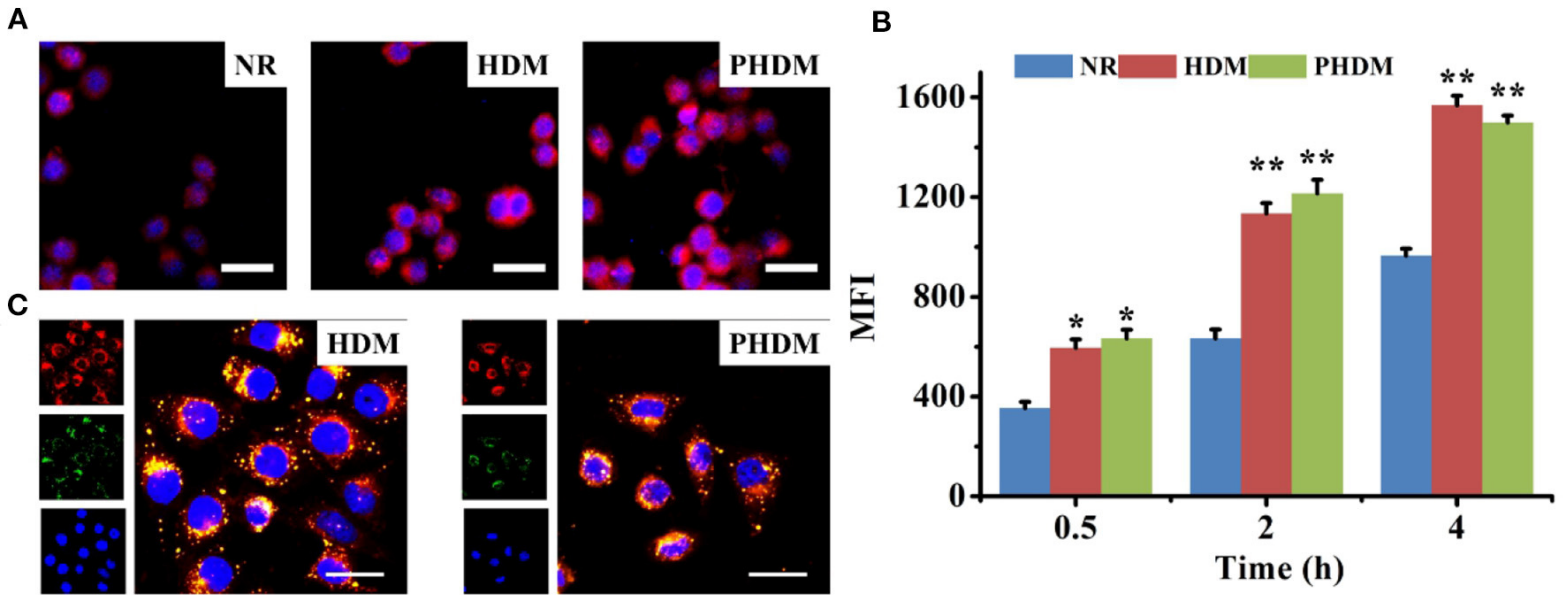
**(A)** CLSM observation of cellular uptake of different formulations for 4 h, red and blue fluorescence indicated formulations and nucleic, scale bars represented 100 μm; **(B)** flow cytometry determines cellular uptake of different formulations at interval time points; **(C)** intracellular drug release behavior of HDM and PHDM, red, green, and blue fluorescence indicated formulations, endo/lysosomal and nucleic, scale bars represented 50 μm.

CLSM and flow cytometry were both used for cellular uptake assay. As shown in Figure 4A, cellular uptake of NR possessed moderate red fluorescence, indicating the uptake capability of the free drug was limited. In comparison, both HDM and PHDM possessed significantly higher red fluorescence, suggesting both formulations could efficiently accumulate into cytoplasm *via* the CD44 receptor-mediated endocytosis. Flow cytometry results (Figure 4B) possessed all formulations, possessed time-dependent uptake capability, and similar uptake trends as CLSM.

In order to investigate intracellular drug release behavior, LysoTracker Green was used to stain endo/lysosomes, and different formulations were incubated with cells. As shown in Figure 4C, yellow pixel dots indicated co-localization of formulations and nucleic, which indicated the formulations were accumulated into endo/lysosomes. For both DHM and PHDM, significant red fluorescence could be observed in the image, which indicated drug was successfully released into cytoplasm.

### *In-vivo* Biodistribution and Antitumor Activity

**Figure 5:**
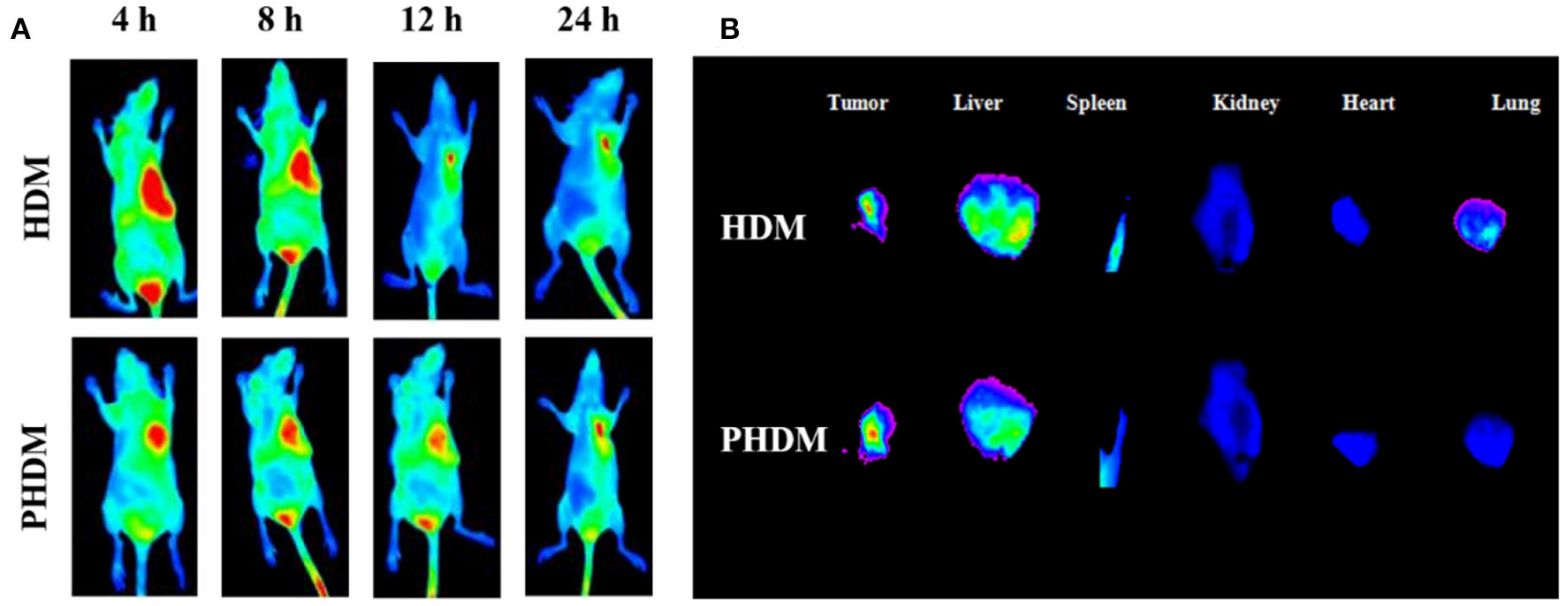
*In-vivo* biodistribution of HDM and PHDM in B16-tumor bearing mice **(A)** and biodistribution in major organs after 24 h administration **(B)** with IVIS system.

*In-vivo* biodistribution and antitumor activity of different formulations were investigated using tumor-bearing mice. DiR was used as fluorescence probe to detect the biodistribution of different formulations. As shown in Figure 5, when intravenous administrated of HDM and PHDM for 4 h, significant tumor targetability of both formulations could be observed with strong fluorescence in the tumor. With an increase of circulation time, the fluorescence decreased gradually and at 24 h, and PHDM possessed relative higher fluorescence in tumor sites. This phenomenon was mainly attributed to two reasons: (Wu et al., 2016) PHDM owes higher stability, which could release drug leakage; and (Zheng et al., 2017) due to the presence of F68, PHDM owes relative loner circulation time. All these results indicated PHDM is a suitable carrier for tumor-targeted chemotherapeutical agent delivery, and it could be used for further study.

**Figure 6:**
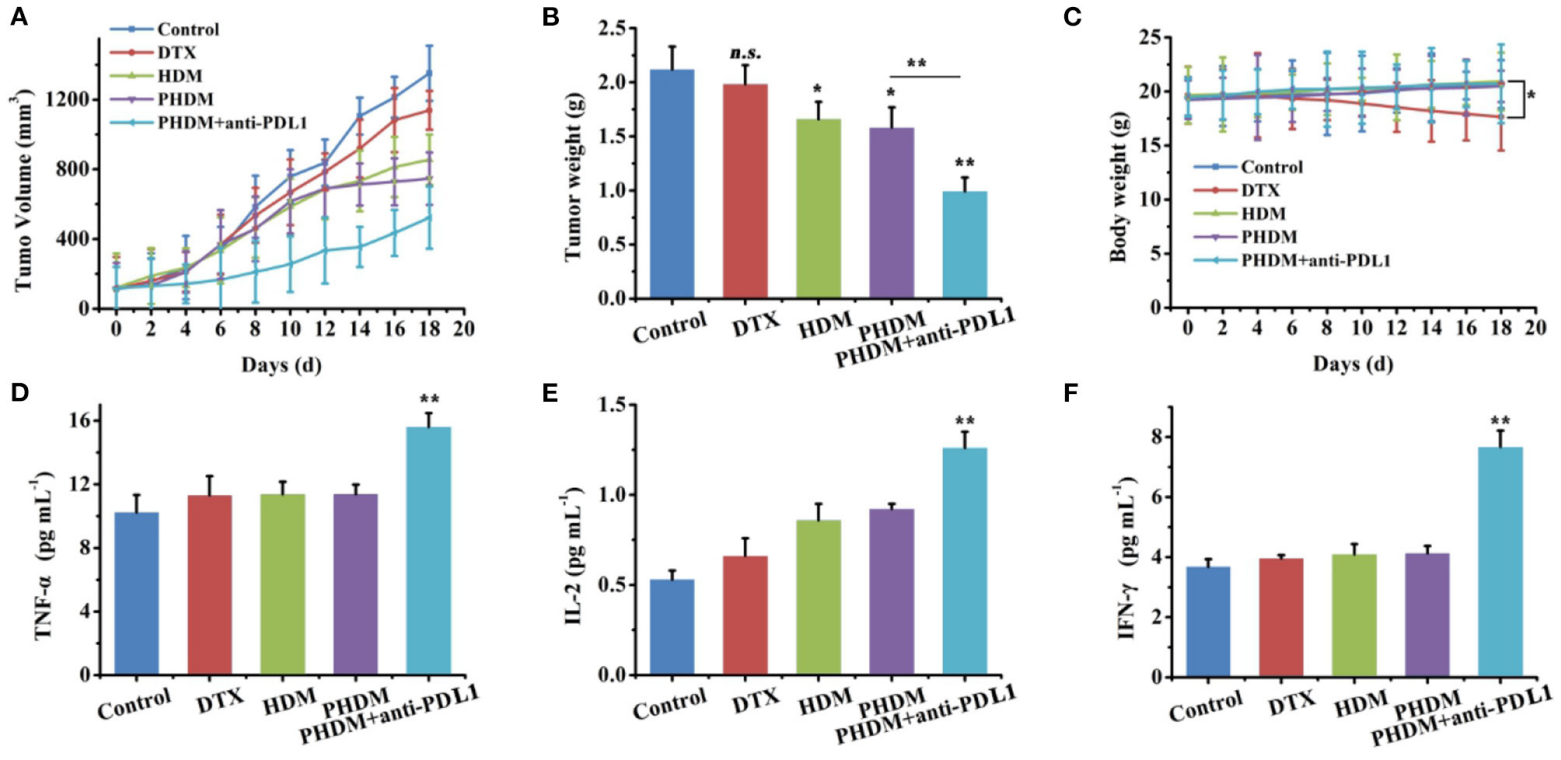
*In-vivo* antitumor activity of different formulations against B16-tumor bearing C57 mice. Tumor volume **(A)**, tumor weight, **(B)** and body weight changes, **(C)** of different formulation groups, **(D–F)** serum cytokines measurement using ELISA kits.

*In-vivo* antitumor activity of different formulations was investigated using B16-bearing C57BL/6 mice. As shown in Figures 6A–C, compared with control group, DTX only showed limited antitumor activity, and HDM and PHDM possessed higher antitumor activity. This result was mainly because the free drug possessed limited tumor targetability, which showed limited antitumor activity. However, both formulations could accumulate to tumor sites *via* the EPR effect and target tumor cell *via* HA-mediated endocytosis. PHDM + anti-PDL1 possessed the best antitumor activity, and this result was mainly because the introduction of the antibody could not only activate immune checkpoint-based immunotherapy, but also enhance the sensitivity of DTX-based chemotherapy. For the body weight study, only DTX possessed significant body weight loss, which was mainly attributed to the non-specific distribution and severe side effects of free DTX.

### Cytokines Measurement and Immune Cell Analysis

In order to further demonstrate whether PHDM+anti-PDL1 achieve tumor immune-chemotherapy, a series of T cell cytokines was measured. As shown in Figures 6D–F, IFN-γ,TNF-α, and IL-2 play an active role in the antitumor immune response, which was associated with immune activation. Significant increase for these cytokines of PHDM+anti-PDL1 group was compared with other groups, which indicated this strategy could relieve immunosuppression and activate immune system for tumor immunotherapy.

**Figure 7:**
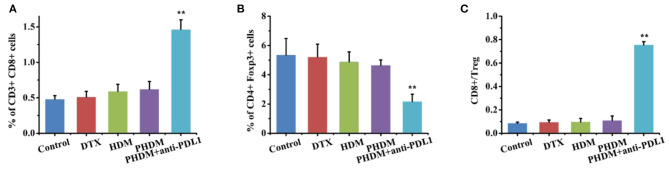
Flow cytometry detection of immune cell population with cocktail antibodies. **(A)** CD8^+^ T cells; **(B)** Treg cells; **(C)** CD8^+^/Treg ratio.

Subsequently, CD8^+^ T cell and regulatory T cells were both measured by flow cytometry. As shown in Figure 7, the percentage of CD3^+^CD8^+^ cells increased, CD4^+^Foxp3^+^ cells decreased, and the ratio of CD8+/Treg increased for PHDM+anti-PDL1. It is well-acknowledged that tumor tissue possessed an immunosuppressive microenvironment and inactive effector T cells from PD-1/PD-L1 axis, which is the main reason for the failure of tumor therapy. Although a chemotherapy-based drug-delivery system could directly kill tumor cells *via* cytotoxic drugs, the clinical benefit is unsatisfied. In this study, the combination of chemotherapy and PD-L1 antibody is a suitable strategy for better antitumor therapy. The PD-L1 antibody could block the PD-L1 protein in tumor cells, which sends the “don't eat me” signal to T cells and induces a T-cell-based antitumor immune response. The released effector cytokines could enhance this process for long-term tumor progression inhibition. Meanwhile, PHDM could efficiently target tumor cells and intracellularly deliver antitumor molecules for tumor killing. All these results indicated that the combination therapy strategy could efficiently activate CD8^+^ T cells and decrease Treg to relieve tumor immunosuppression and activate the antitumor immune response.

## Conclusion

Overall, in this study we constructed a DTX-loaded HA-based copolymer-mixed micelles in combination with PD-L1 antibody for tumor immune-chemotherapy. The as-synthesized HA-VES could be efficiently entrapped hydrophobic molecule DTX, and F68 could be used for mixed micelle construction. After injection, PHDM could efficiently accumulate into tumor sites and target tumor cells for tumor chemotherapy. While the PD-L1 antibody could also bind tumor cells for immune checkpoint inhibition for tumor immunotherapy, all the results demonstrated that PHDM+anti-PDL1 was a potential for tumor immune-chemotherapy.

## Data Availability Statement

The raw data supporting the conclusions of this article will be made available by the authors, without undue reservation.

## Ethics Statement

The animal study was reviewed and approved by Experimental Animal Administrative Committee of Jiamusi University.

## Author Contributions

CZ and XD: perform the experiment and write manuscript. CS and SC: performance the data analysis. TC and DZ: perform data analysis. XZ and CY: contribute to the conception of the study. All authors contributed to the article and approved the submitted version.

## Conflict of Interest

The authors declare that the research was conducted in the absence of any commercial or financial relationships that could be construed as a potential conflict of interest.
