# Supplementary material for: Rational Design of Hyaluronic Acid-Based Copolymer-Mixed Micelle in Combination PD-L1 Immune Checkpoint Blockade for Enhanced Chemo-Immunotherapy of Melanoma
Source: Front Bioeng Biotechnol. 2021 Mar 10;9:653417. doi: 10.3389/fbioe.2021.653417 (PMC7987940; doi:10.3389/fbioe.2021.653417)
Supplement: Supplementary file 1 [file Image_1.pdf]

Supporting Information for

Rational design of hyaluronic acid based copolymer mixed micelle in combination PD-L1 immune checkpoint blockade for enhanced chemo-immunotherapy of melanoma

Chaopei Zhou[1#], Xiuxiu Dong[1#], Chunxiang Song[1], Shuang Cui[1], Tiantian Chen[1], Daji Zhang[1], Xiuli Zhao[2], Chunrong Yang[1]*

[1]*College Pharmacy, Jiamusi University, 258 Xuefu Street, Jiamusi, Heilongjiang 154007, China*

[2]*School of pharmacy, Shenyang Pharmaceutical University, Shenyang 110016, P.R. China*

Chaopei Zhou[1#], Xiuxiu Dong[1#] contribute equal to this work

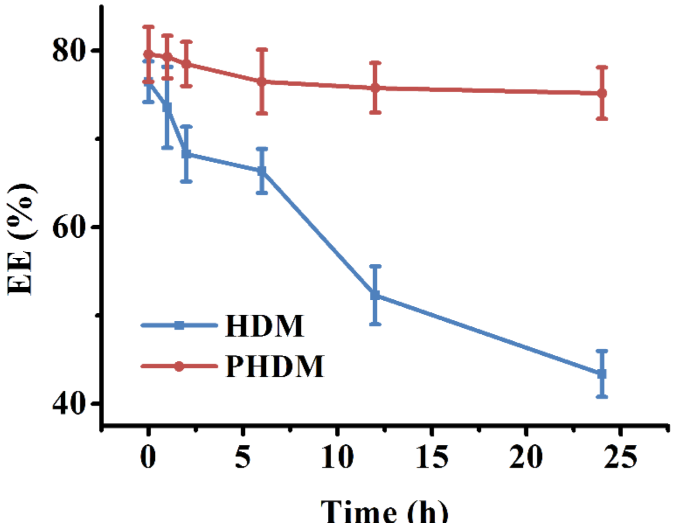

Fig.S1: *in vitro* stability of HDM and PHDM with EE% as a parameter.
